# Relationship among *Streptococcus gallolyticus* Subsp. *gallolyticus*, *Enterococcus faecalis* and Colorectal Neoplasms in Recurrent Endocarditis: A Historical Case Series

**DOI:** 10.3390/jcm11082181

**Published:** 2022-04-13

**Authors:** Eva Romay, Juan Manuel Pericàs, María José García-País, Marta Hernández-Meneses, Blanca Ayuso, Javier García-González, Rodrigo Vicente Garcés-Durán, Ramón Rabuñal, Pilar Alonso-García, Fernando García-Garrote, Andrés Perissinotti, Bàrbara Vidal, Carles Falces, Eduard Quintana, Leticia Moreira, Manel Almela, Josep Llach, Asunción Moreno, Juan Corredoira, Jose María Miró, on behalf of Lucus Augusti and Hospital Clinic Endocarditis Teams

**Affiliations:** 1 Infectious Diseases Department, Hospital Lucus Augusti, 27003 Lugo, Spain; eva.maria.romay.lema@gmail.com (E.R.); maria.jose.garcia.pais@sergas.es (M.J.G.-P.); blanca.ayuso-garcia@sergas.es (B.A.); ramon.rabunal.rey@sergas.es (R.R.); pilar.alonso.garcia@sergas.es (P.A.-G.); fernando.garcia.garrote@sergas.es (F.G.-G.); juan.corredoira.sanchez@sergas.es (J.C.); 2 Infectious Diseases Department, Hospital Clínic de Barcelona, Institut d’Investigacions Biomèdiques Pi i Sunyer, University of Barcelona, 08036 Barcelona, Spain; jpericas@clinic.cat (J.M.P.); mhmeneses@clinic.cat (M.H.-M.); jagarcia@clinic.cat (J.G.-G.); amoreno@clinic.cat (A.M.); 3 Liver Unit, Internal Medicine Department, Vall d’Hebron University Hospital, Vall d’Hebron Institute for Research (VHIR), 08036 Barcelona, Spain; 4 Centro de Investigación Biomédica en Red de Enfermedades Hepáticas y Digestivas (CIBERehd), 28029 Madrid, Spain; 5 Gastroenterology Service, Hospital Clínic de Barcelona, Centro de Investigación Biomédica en Red de Enfermedades Hepáticas y Digestivas (CIBERehd), Institut d’Investigacions Biomèdiques Pi i Sunyer, University of Barcelona, 08036 Barcelona, Spain; rvgarces@clinic.cat (R.V.G.-D.); lmoreira@clinic.cat (L.M.); jllach@clinic.cat (J.L.); 6 Nuclear Medicine Department, Hospital Clínic de Barcelona, Institut d’Investigacions Biomèdiques Pi i Sunyer, Biomedical Research Networking Center of Bioengineering, Biomaterials and Nanomedicine (CIBER-BBN), 08036 Barcelona, Spain; aperissi@clinic.cat; 7 Cardiology Department, Hospital Clínic de Barcelona, Institut d’Investigacions Biomèdiques Pi i Sunyer, University of Barcelona, 08036 Barcelona, Spain; bvidal@clinic.cat (B.V.); cfalces@clinic.cat (C.F.); 8 Cardiovascular Surgery Department, Hospital Clínic de Barcelona, Institut d’Investigacions Biomèdiques Pi i Sunyer, University of Barcelona, 08036 Barcelona, Spain; equintan@clinic.cat; 9 Microbiology Department, Hospital Clínic de Barcelona, Institut d’Investigacions Biomèdiques Pi i Sunyer, University of Barcelona, 08036 Barcelona, Spain; malmela@clinic.cat

**Keywords:** infective endocarditis, *Enterococcus faecalis*, *Streptococcus gallolyticus*, recurrences, reinfections, colorectal neoplasms, colonoscopy

## Abstract

Objectives: The role of colorectal neoplasms (CRN) as a common potential source of recurrent *Streptococcus gallolyticus* subsp. *gallolyticus* (SGG) and *Enterococcus faecalis* (EF) endocarditis remains unstudied. We aimed to investigate what proportion of episodes of recurrent endocarditis are caused by a succession of SGG and EF, or vice versa, and to assess the role of a colonic source in such recurrent episodes. Methods: we conducted a retrospective analysis of two prospective endocarditis cohorts (1979–2019) from two Spanish hospitals, providing descriptive analyses of the major features of the endocarditis episodes, colonoscopy findings, and histologic results. Results: among 1552 IE episodes, 204 (13.1%) were caused by EF and 197 (12.7%) by SGG, respectively. There were 155 episodes (10%) of recurrent IE, 20 of which (12.9%) were due to a succession of SGG/EF IE in 10 patients (the first episode caused by SGG in eight cases, and by EF in two cases). The median follow-up was 86 (interquartile range 34–156) months. In 8/10 initial episodes, the causative microorganism was SGG, and all patients were diagnosed with CRN either during the initial episode or during follow-up. During the second episode of IE or follow-up, colonoscopies revealed CRN in six patients. Conclusions: There seems to be an association between SGG and EF in recurrent endocarditis that warrants further investigation. Our findings reinforce the need for systematically performing colonoscopy in the event of endocarditis caused by both microorganisms.

## 1. Introduction

Approximately 2–6% of patients with infective endocarditis (IE) will have one or more recurrent episodes of IE throughout their lives [1]. When these recurrent episodes occur shortly after the initial episode (generally within six months) and are caused by the same microorganism, it is considered a relapse. When recurrent IE are caused either by a microorganism other than the one causing the initial episode, or by the same micro-organism, but more than six months later, it is considered a re-infection. Ideally, molecular studies should be carried out to rule out strains of the microorganism belonging to the same clone [2]. In a study by the International Collaboration on Endocarditis, Alagna and colleagues identified intravenous drug use and hemodialysis as chief factors associated with re-infection [1], whereas a gastrointestinal tract focus, including invasive gastrointestinal procedures, is currently considered a minor cause of re-infection [1,3].

*Enterococcus faecalis* (EF) and *Streptococcus gallolyticus* subsp. *gallolyticus* (SGG, formerly known as *S. bovis* I) are frequent causes of IE, especially in elderly patients [4,5,6]. These microorganisms share phenotypic characteristics (both were formerly taxonomically grouped as D group streptococci) and a common habitat: the intestine. The association of SGG with colorectal neoplasms (CRN) is very well established [7]. More recently, the association of EF with colonic tumors has also been described, and several experts argue that systematically performing colonoscopy after EF bacteremia/IE should become common practice, as in the case of SGG [5,6,8,9,10].

The origins of EF IE are poorly known. In some cases, a possible gateway is identified in urinary manipulation, catheter infection, etc., but in most cases, the primary focus is unknown [6]. In these cases, the primary source likely lies in the gut, but our knowledge is still in its infancy when it comes to the underlying mechanisms, including the role played by CRN, in its genesis. These mechanisms are better known in the case of SGG IE, in which an extraintestinal focus is not usually detected, and strong evidence suggests that CRN plays a decisive role [11,12]. In available studies, the presence of CRN has not been shown to be associated with a higher likelihood of relapse, either for SGG and EF IE [5,6,8,10,13,14,15,16]. Seemingly, the two microorganisms might also potentially share common pathways in the pathophysiology of bloodstream infections, such as IE, and even cause successive episodes of IE in patients with predisposing factors at the gut level. However, the occurrence of relapsing IE episodes by both microorganisms has not been assessed thus far.

We aimed to analyze IE recurrences in patients who had suffered IE episodes by both microorganisms and to explore the potential role of the colorectal tract in the development of such recurrent IE episodes.

## 2. Materials and Methods

### 2.1. Design

Retrospective analysis of two cohorts collected through a prospective registry in two Spanish university hospitals: Hospital Clínic, Barcelona (HCB), with 830 beds, a referral center for cardiac surgery, and Lucus Augusti University Hospital, Lugo (HULA), with 690 beds, and without cardiac surgery facilities. The collection periods were: 1979–2019 (HCB) and 1987–2019 (HULA).

### 2.2. Patients

Patients with recurrent definite IE caused consecutively by SGG and EF, and vice versa, were considered for this study. Only the first episode of recurrence was analyzed in those patients with more than one recurrence after the first episode of IE. All patients had at least one-year follow-up after the initial IE episode. Colonoscopies were performed following the international guidelines for SGG IE (i.e., carried out systematically), whereas it was performed at the discretion of the treating physician for EF IE.

### 2.3. Definitions

The diagnosis of IE was made according to the modified Duke criteria [17]. We analyzed only episodes of reinfection where either SGG IE followed EF IE, or vice versa. CRN included adenomas and carcinomas. Advanced adenomas were defined as those fulfilling at least one of the following criteria: ≥1 cm diameter, tubulovillous (25–75% villous component) or villous (>75%) histology, high-degree dysplasia, or ≥3 adenomas. In situ carcinoma was considered as a high-degree dysplastic adenoma. Invasive carcinoma was defined as that in which malign cells were found beyond the muscularis mucosa [5].

### 2.4. Ethics

The ethical review boards of both institutions approved the study. All patients provided informed consent.

### 2.5. Variables and Analyses

The variables include the description of patients’ baseline characteristics and major comorbidities, causative microorganism, characteristics of both first and recurrent IE episodes (date, valve/s affected (native or prosthetic), severe complications), performance of colonoscopy, endoscopic and histological findings, follow-up, deaths during follow-up, and causes of death. Results are expressed as medians (interquartile range, IQR) and percentages. All statistical analyses were carried out using SPSS software, v20 (Chicago, IL, USA).

## 3. Results

During the study period, 1552 IE episodes were diagnosed across both sites, 204 (13.1%) and 197 (12.7%) of which were caused by EF and SGG, respectively. There were 30 relapses (1.9%) and 125 re-infections (8%). At HCB, EF predominated over SGG (157 vs. 83), whereas SGG predominated (114 vs. 47) at HULA. Twenty-three (11.3%) and eighteen (9.1%) patients with EF IE and SGG IE, respectively, presented at least one episode of re-infection. In 10 of these cases (24.4%), the same patient presented consecutive IE caused by SGG and EF. It was these 10 patients with their 20 episodes of IE that were analyzed. The first episode was caused by SGG in eight cases and by EF in two cases.

The median age during the first episode was 69.5 years (IQR 65–79 years) and eight (80%) were male. Almost all had comorbidities (Table 1).

The presentation was subacute, with 26.3 days (IQR 12–47) as the median length of symptoms prior to admission. Only one patient presented symptoms suggestive of intestinal pathology (Case 6, during the second episode of IE). Six episodes (30%) were native IE and 14 prosthetic IE (70%). The aortic valve was involved in 10 cases (50%), the mitral valve was affected in seven (35%), and mitro-aortic involvement was identified in three cases (15%). Three episodes (15%) were complicated with spondylitis.

The median time elapsing between the first and second IE episodes was 23.5 months (IQR 13–50 months), with 7/10 episodes occurring within three years.

Valve replacement was carried out in four (20%) episodes (two during the IE episode, and within 2 years from the initial episode in the other cases). In another patient, an infected pacemaker was removed. Eighteen episodes (90%) ended in cure, whereas two patients died of heart failure during the second episode (Cases 6 and 10, Table 1).

The median follow-up was 86 months (IQR 34–156). During follow-up, two patients presented new episodes of IE caused by SGG, which were not subjected to molecular studies to determine whether the same clones were causal. In one case, the SGG IE recurrence occurred four years after the first SGG IE and was interpreted as a re-infection (case No. 4, Table 1). The second case occurred in a patient who presented three further episodes of IE over a three-year period, which were interpreted as relapses, since the infected pacemaker was not removed (Case 7, Table 1). Two patients also presented recurrences due to viridans group streptococci. During follow-up, five patients (25%) died: three due to cancer-related causes (none with colorectal cancer) and two due to chronic obstructive pulmonary disease complications. 

### Colon Studies

During the first episode of IE, a colonoscopy was performed in 8/10 patients (all caused by SGG), in six (60%) of which CRN were found (all advanced adenomas, including two in situ carcinomas). In the other two cases (both caused by EF), colonoscopy could not be performed due to the patients’ poor clinical condition during the IE episode. However, in one case, a colonoscopy had been performed three years earlier, and in the other case, a colonoscopy was performed seven months after the episode, both of them showing advanced adenomas (Table 1).

During the second episode of IE, colonoscopies were performed on six patients, finding adenomas in five (four of them advanced). Multiple adenomas were found in a subsequent colonoscopy performed on the patient in whom no lesions were demonstrated (poor preparation in two colonoscopies) during the IE episode. In another patient on whom colonoscopy could not be performed, an intestinal obstruction caused by a colorectal tumor was found and not removed, given a high surgical risk (Case 6, Table 1). In all other patients, all colonic neoplasms were removed during the colonoscopy. Two of the three patients on whom colonoscopy was not performed during the second IE episode died of cancer (gastric and leukemia, respectively). Colonoscopies were performed on four patients during follow-up after the second episode of IE, detecting new adenomas (all advanced) in three of them (Table 1). None of the patients died due to colorectal cancer during follow-up.

## 4. Discussion

Our study provides the basis to hypothesize that SGG and EF share not only the colon as the main source of infection, but also common pathophysiological mechanisms related to the disruption of the colonic wall, particularly due to CRN. This is of relevance, as both microorganisms are major causes of IE in Western countries and, in particular, the incidence of EF IE is increasing [4]. Moreover, both are chief causes of recurrent IE [1,14].

In most cases of EF and SGG IE, an apparent focus of infection is not detected [6]. However, the colon is not systematically explored in initial episodes of EF IE and only seldomly in recurrent EF and SGG IE episodes [1]. The generally silent finding of intestinal pathology leads us to suspect that it is the possible focus of bacteremia, while the role of benign colorectal lesions in the genesis of bacteremia remains doubtful. Several studies have described such benign colonic lesions (e.g., diverticula, hyperplastic polyps, angiodysplasia) in a notable proportion of patients with EF and SGG IE undergoing colonoscopy [6,8,10]. Nonetheless, we found no significantly higher incidence of benign colonic lesions in patients with SGG bacteremia/IE than in the general population in a case-control study [15].

In the present study, CRN were detected in 77% of cases in which colonoscopy was performed during the IE episode, as well as in 89% of colonoscopies performed during follow-up. These figures are slightly higher than in prior studies, which reported CRN in 50–75% of patients with IE due to EF or SGG undergoing a colonoscopy, which is much higher than in the age- and sex-matched general population [5,6,7,8,9,10,15,16]. Clinical data suggest that SGG plays a relevant role in the early stages of tumor development, even in the absence of the adenomatous polyps that allow SGG to colonize the colon and subsequently cause bloodstream infections. In approximately 30% of SGG IE, no CRN is found during colonoscopy [15]. Nonetheless, a notable proportion of patients with SGG IE and no CRN in the initial colonoscopy do develop CRN over time [5,13]. The same applies to patients with SGG IE that do present a CRN in the initial colonoscopy. In summary, periodic follow-up colonoscopies are mandatory in all patients with SGG IE for the rest of their lives, irrespective of the results of the initial colonoscopy [5,13].

In 8/10 patients in this series, the IE caused by SGG preceded the IE episode caused by EF. In a prior study, we found that the percentage of patients with SGG IE with no further IE episodes that presented CRN was 71.4% (55/77) [14], similar to that of patients with SGG IE presenting recurrent IE in the present series (5/8, 62.5%). Despite the available evidence pointing to a potentially more relevant role of SGG in subsequently diagnosed CRN [5,17], given the complex interactions between different members of the colonic flora in the development of cancer, it is still insufficient to sustain this hypothesis. Future studies should analyze in detail the genomic and phenotypic features, including the oncogenic properties, of the SGG and EF strains isolated from patients with CRN and compare them to those isolated from patients without CRN. Likewise, detailed studies are needed on the changes in the colonic microbiota associated with CRN, especially in its early stages, with a view to clarifying the roles and temporal sequencing of the range of potentially oncogenic bacterial species, including the host response and susceptibility. It should be noted, however, that investigating potential gut bacteria associations over time is challenging, as temporal changes of microbiota are frequent, even in normal physiological conditions (e.g., induced by diet, antibiotics, or aging).

Our study has several limitations. It has a retrospective design lacking controls. Furthermore, a potential historical bias might have affected routine practices regarding the performance of colonoscopies, particularly in EF IE cases. In addition, the two participating centers do not share organizational and epidemiological characteristics, potentially impacting the profiles and prevalence of SGG/EF IE at each site: HCB is a large reference center for cardiac surgery, serving a mostly urban area, whereas HULA is not a cardiac reference center and serves a mostly rural area.

## 5. Conclusions

EF and SGG are major causes of recurrent IE, and they might present in association. The high prevalence of CRN in patients with EF and SGG IE suggests damaged colonic mucosa as a relevant predisposing factor for recurrent IE. Our findings reinforce the need for systematically performing colonoscopy in patients with EF and SGG. Further studies should characterize the underlying mechanisms of the apparent synergistic relationships among EF, SGG, CRN, and IE.

## Figures and Tables

**Table 1:** Characteristics of 20 recurrent episodes of endocarditis caused by either *Enterococcus faecalis* or *Streptococcus gallolyticus* subsp. *gallolyticus* in 10 patients.

First IE Episode					Second IE Episode				
Patients	Date (M/Y)	Age/sex, comorbidities	Type of IE, microorganism, complications	Colonos-copy *	Date (M/Y)	Type of IE, micro-organism, complica-tions	Colonoscopy	Follow-up, status	Comments
Case 1	8/2002	68/M, DM, CKD.	NVE Ao EF.	Not performed (due to embolic stroke)	3/2003	PVE Ao SGG Spondylitis	Tubular adenomas (4, 2 of them > 1 cm)	8/2015, deceased (COPD)	2007, 2009, 2010, and 2013 advanced adenomas
Case 2	6/2004	67/M, IHD, COPD	PVE Ao SGG	Tubulovillous adenoma	2/2011	NVE M EF	Tubular adenomas (9)	8/2012, deceased (COPD)	
Case 3	7/2008	73/M Renal and prostate cancer	NVE Ao SGG	Tubular adenomas (8), radiation proctitis	11/2009	NVE Ao EF	Tubular adenomas (3)	11/2020 alive	4/2017 *S. sanguis* IE; 2013, 2017, and 2020 advanced adenomas
Case 4	4/2004	71/M DM, IHD, renal carcinoma.	NVE M-Ao SGG Spondylitis	Villous adenoma	6/2008	PVE Ao EF	Villous adenoma	6/2010, deceased (renal carcinoma)	8/2008 SGG IE; 3/2009 *S. salivarius* IE
Case 5	6/2016	83/M DM, acute myeloid leukemia, COPD, CKD.	PVE Ao EF	Not performed (leukemia)	7/2017	NVE M, PVE Ao SGG	Not performed (leukemia)	10/2018, deceased (leukemia)	2002 EF IE T013 tubulovillous adenoma
Case 6	1/2011	80/F DM, PCM.	PVE M-Ao SGG	Tubular adenoma with high-grade dysplasia	4/2011	PVE Ao EF	Not performed (neoplastic intestinal occlusion)	5/2011, deceased during IE episode (heart failure)	
Case 7	10/2011	65/M Liver cirrhosis, IHD, PCM.	PVE Ao SGG	Ischemic colitis	2/2013	PVE EF	Not performed (heart failure)	6/2015, deceased (new SGG IE)	Four SGG IE episodes in total (2011, 2012, 2013, 2015); 9/2014 Gastric cancer
Case 8	2/2005	53/M DM, COPD.	NVE M SGG	Tubular adenomas (4)	10/2007	PVE M EF Spondylitis	Normal (poor preparation in two occasions)	10/2020 alive	4/2012 Multiple polyps, not biopsied; 2/2013 Tubular adenomas (3); 1/2020 Tubular adenomas (7)
Case 9	8/2007	57/M CHD.	PVE M SGG	In situ carcinoma and tubulovillous adenomas (8)	10/2011	PVE M EF	Tubular adenoma	08/2020 alive	2003 *S. mitis* IE 6/2020 normal colonoscopy
Case 10	3/2011	79/F DM, IHD, CKD.	PVE M SGG	Normal	10/2013	PVE M EF	Not performed (volvuli in sigma)	1/2014 deceased during IE episode (heart failure)	

* Number of lesions between brackets; Ao: aortic; CHD: congenital heart disease; CKD: chronic kidney disease; COPD: chronic obstructive pulmonary disease; DM: type II diabetes mellitus; EF: *Enterococcus faecalis*; M: male; F: female; IE: infective endocarditis; IHD: ischemic heart disease; M: mitral; M-Ao: mitro-aortic; NVE: native valve endocarditis; PCM: pacemaker; PVE: prosthetic valve endocarditis; SGG: *S. gallolyticus* subsp. *gallolyticus*.

## Data Availability

Data are available upon request.
